# The Twitter pandemic: The critical role of Twitter in the dissemination of medical information and misinformation during the COVID-19 pandemic

**DOI:** 10.1017/cem.2020.361

**Published:** 2020-04-06

**Authors:** Hans Rosenberg, Shahbaz Syed, Salim Rezaie

**Affiliations:** * Department of Emergency Medicine, University of Ottawa, Ottawa, ON; † Department of Emergency Medicine, Greater San Antonio Emergency Physicians, San Antonio, TX

**Keywords:** COVID-19, pandemic, Twitter

As the world finds itself in the middle of the COVID-19 pandemic, social media has become inundated with content associated with the virus. Although all social media platforms (e.g., Facebook, Instagram, blogs) are currently providing us with medical content, perhaps no other consistently plays a more prominent role in the medical world than Twitter.^1^ Emergency medicine (EM) is on the bleeding edge, where practice at the bedside is continually being shared on social media and this pandemic has resulted in immense activity on Twitter.

Twitter is a microblogging and social networking service where users post messages using “tweets” that are limited to 240 characters. For well over a decade, Twitter has become increasingly used as a platform where medical practitioners exchange ideas, information, and commentary. The hashtag #FOAMed garners thousands of tweets per hour, and at this momentous period in medical history, no subject is more prominent than COVID-19. With the free-flow of messages and ideas that are not vetted or peer-reviewed, unlike classic medical educational resources, is there a risk of harm? What are the benefits to the EM community from Twitter? Finally, how does the average emergency physician (EP) get the most out of the information out there?

## HARMS

Hysteria. You cannot be on Twitter and paying attention to the pandemic without noticing multiple posts declaring this is the apocalypse. Although tongue in cheek for some, there is a hashtag #apocalypse2020 for all those who are preparing for the end. This is risky for many reasons and, very importantly, the mental well-being of Twitter users. Social media have been associated with increased mental distress, self-harm, and suicide.^2^ Additionally, the spread of information is not limited by distance, such that the pandemic of fear can and has spread before the actual C-19 pandemic.^3^ This has an effect on our patients’ mental health, putting their physical and mental well-being at risk. This can lead to an increase in suicidal ideation or attempts and is something that the EP has to prepare for at increasing rates during this pandemic. Let's not forget the similar risk to frontline medical providers, being exposed to the same stresses and endangering our mental well-being.

Social media platforms are well known for the spread of misinformation and denial of scientific literature. Perhaps, no example is more prominent than the rise of vaccine hesitancy and the role that social media play in the spread of inaccurate and negative information.^4^ The current COVID-19 pandemic is not immune to this misinformation and, in fact, is the “first social media pandemic.”^5^ Few examples of misinformation are more obvious than U.S. President Donald J. Trump's tweet touting the combination of hydroxychloroquine and azithromycin as one of the “biggest game changers in the history of medicine.”^6^ This, of course, turned out to be a falsehood with multiple respected medical bodies asking for physicians to keep from prescribing the combination.^7^ Whether well-intentioned or malicious in nature, the spread of misinformation leads to fear, inappropriate prescribing, less response to warnings on issues such as social-distancing, and mistrust in the medical advice due to the plethora of misinformation.

**Figure 1.**
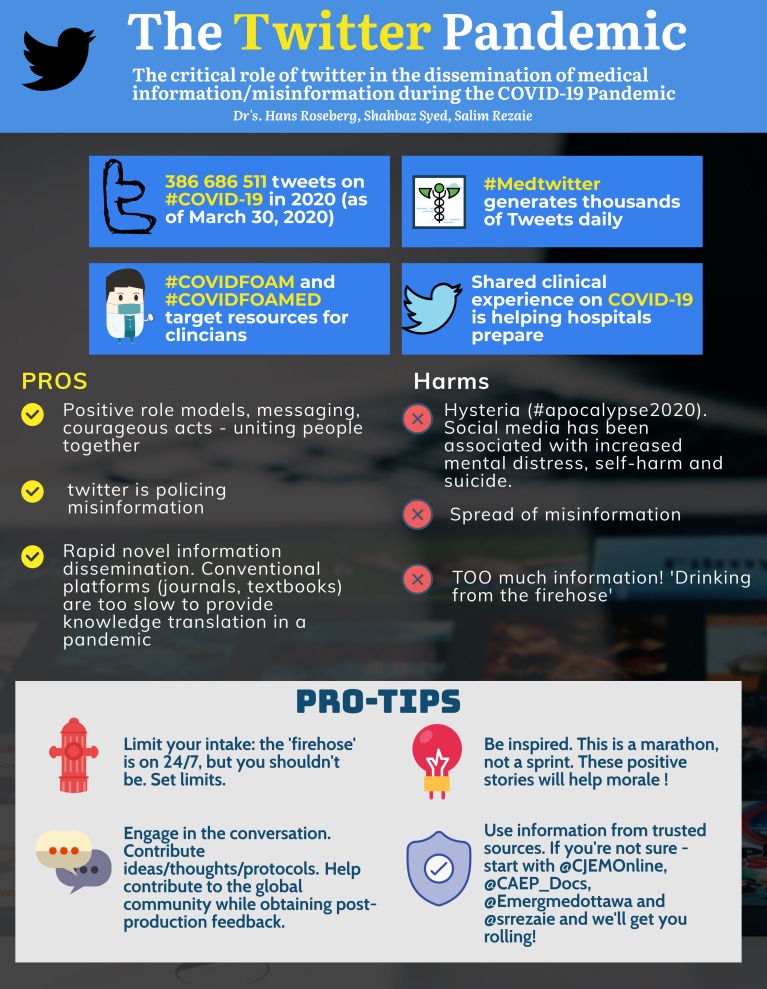
Infographic – Twitter pandemic.

A deluge of information. There really is no other way to describe the amount of information being microblogged about COVID-19. This can be a good thing, but the massive amount of information can be overwhelming to the end-user. One of the most commonly cited reasons people do not use platforms such as Twitter is the sheer amount of information.^8^ As Choo et al. described it, “drinking from a firehose” of information becomes a barrier to education, whether it relates to COVID-19 pandemic or other topics.

## BENEFITS

Fortunately, there are multiple positives to Twitter in the age of the COVID-19 pandemic.

It allows users to be inspired by stories of courageous acts, positive role models, and global efforts to combat the pandemic. From frontline health care workers with their tales of unimaginable sacrifices, to the non-medical users simply surviving through a quarantine period with some humor, these are the type of stories that can help people get through this crisis together. One of these inspiring examples is Dr. Yale Tung Chen, an EP who chronicled his COVID-19 infection, including ultrasound findings,^9^ and simultaneously demystifying the illness while educating the world in a novel format.

To counter concerns regarding misinformation and the validity of claims being made, Twitter is actively fighting to stop the misinformation or damage that may come to users from posts on their platform.^10^ They are actively removing tweets with content that denies global or local health authority recommendations to decrease someone's likelihood of exposure, description of treatments that are harmful or not immediately harmful but are known to be ineffective, content that denies facts about transmission, and claims impersonating official government agencies. These efforts can go a long way in appeasing the real and perceived inaccuracies of the content on the social media platform.

One of the criticisms of conventional medical education (textbooks, journals) is that they are often behind the curve in terms of knowledge translation. The benefit of social media is that the content is often more novel. In the middle of a pandemic, the ability to rapidly share information is critical for knowledge translation and dissemination, and Twitter is able to do this in a way that is typically not feasible for textbooks or journals. Most relevant to the EM community, we are seeing the free exchange of protocols/guidelines from specialty groups such as the Canadian Association of Emergency Physicians, highly reputable international journals (such as *Journal of the American Medical Association*, *New England Journal of Medicine*, *BMJ* [formerly *British Medical Journal*], and others), and academic/community hospitals. Leading on this front are many of the established medical educators who use Twitter as part of the Free Online Access Medical Education (FOAMed) effort to educate EPs on the global pandemic. Just one of these examples is the “Protected Airway Process” from Dr. Christopher Hicks, widely shared and retweeted by frontline practitioners to ensure that health care providers remain safe during intubations.^11^ It's amazing to think that medical professionals everywhere around the globe have the same database of information with the simple use of a website or mobile application. Although the user must remain vigilant in the face of any new information presented to them, these respected institutions and users can equip health care workers worldwide with the knowledge to combat the pandemic.

Our advice when using Twitter as a source of information during the COVID-19 global pandemic is the following:

1. Limit your intake. The “firehose” is on 24/7 but you should not be. Set aside some time in the day and stick to that limit. It's a rapidly evolving crisis but not such that it should overwhelm you.
2. Engage in the conversation. Contribute to the discussion or present your ideas/protocols. This form of post-publication feedback may be key to ensuring you are doing what is up to date.
3. Allow yourself to be inspired. This will be a marathon, not a sprint, and you will need these positive stories to keep your spirits up.
4. Ensure you use information from trusted sources. Follow those accounts that truly interest you, unfollow those that don't contribute to your learning. If you are struggling with where to start, follow #COVIDFOAM and #COVIDFOAMED. Consider adding @CJEMonline and @CAEP_Docs to accounts you follow, as we are committed to be the voice of Canadian EPs throughout this pandemic.
5. Most importantly, stay safe.
